# Leonurine affected homocysteine‐methionine metabolism based on metabolomics and gut microbiota studies of clinical trial samples

**DOI:** 10.1002/ctm2.535

**Published:** 2021-10-12

**Authors:** Junyi Liao, Rinkiko Suguro, Xia Zhao, Yue Yu, Yimin Cui, Yi Zhun Zhu

**Affiliations:** ^1^ State Key Laboratory of Quality Research in Chinese Medicine & School of Pharmacy Macau University of Science and Technology Macau SAR China; ^2^ Department of Pharmacy Peking University First Hospital Beijing China; ^3^ Shanghai Key Laboratory of Bioactive Small Molecules Department of Pharmacology School of Pharmacy Fudan University Shanghai China; ^4^ Zhongzhu Pharmaceutics Co. Ltd. Zhuhai China

Dear Editor,

Cardiovascular disease (CVD) remains the leading cause of mortality globally, taking several millions of lives every year. Various risk factors for CVD have been identified over the years. Homocysteine, an amino acid and one of the intermediates in the methionine metabolism, has been closely associated with CVD for decades. Elevated levels of homocysteine are normally associated with raised risk for CVD.^1^ It was suggested that homocysteine could cause endothelial dysfunction, therefore accelerating the formation of thrombin as well as inhibiting native thrombolysis.^2^ Methionine is considered as the precursor of homocysteine in human bodies through multiple reactions. Meanwhile, homocysteine can be converted into methionine, catalyzed by the enzyme, methionine synthase.^3^

Metabolomics provides new interpretations of various physiological processes, by evaluating the levels of various metabolites in biological samples, combined with bioinformatics.^4^ It has become a rising star in the omics studies. Metabolomics has been applied in the studies of CVD and produced novel and significant hypotheses for CVD treatment and prevention.^5^ As for gut microbiota, it has attracted many researchers’ attention in recent years. Gut microbiota, the numerous bacteria that reside in our gastrointestinal tract, contributes greatly to various physiological functions, including contributing to metabolic functions, protecting against pathogens, and educating our immune system.^6^ The dysbiosis of components and abundance in gut microbiota has been associated with various pathological processes.^7^

Leonurine was originally found in the leaves of the traditional Chinese medicine, *Herba Leonuri* (otherwise known as Chinese Motherwort).^8^ It was established in our previous studies that leonurine showed various significant pharmacological effects, including antiapoptotic, antioxidant, and anti‐inflammatory effects. Moreover, leonurine showed a great cardiovascular protective effect against myocardial infarction, myocardial fibrotic response, and atherosclerosis. Long‐term administration of leonurine was reported to improve lipid profiles in various animal models, including mice, rabbits, and rhesus monkeys.^9^ As a promising novel drug, leonurine has now entered clinical trials in China for treating hyperlipidemia. However, the exact mechanism of leonurine's cardioprotective effects was not clearly established.

In this study, we wanted to further explore the effects of leonurine from the angles of metabolomics as well as gut microbiota studies based on the samples collected from leonurine clinical trials, to provide new insights into the mechanisms of leonurine. Clinical samples were collected from participants in the leonurine phase I clinical trial. These candidates were randomly divided into three groups of 12, and in every group, three candidates were randomly assigned to be given the placebo. The three groups of candidates received leonurine treatment at three different dosages, which were 50, 150, and 300 mg. Leonurine treatment was given once daily by oral administration for 7 days. Plasma samples were collected at D1‐0h (right before the first leonurine treatment at Day 1), D7‐0h (right before leonurine treatment at Day 7), and D7‐3h (3 hours after leonurine treatment at Day 7). Fecal samples were collected at D0 (within 24 hours before the first treatment of leonurine) and D7 (within day 7 ± 1).

Since previous clinical experiment has already proved that dosages, even as high as 300 mg per day, showed great safety, we mainly focus on the differences before and after leonurine treatment rather than the dosage differences. Based on liquid chromatography coupled with tandem mass spectrometry, the metabolites in plasma samples were detected and annotated. Partial least square‐discriminant analysis (PLS‐DA) was first applied to access the differences among the three groups. Results suggested that significant differences were observed between D1‐0h and the other two groups (Figure 1A). However, the differences between D7‐0h and D7‐3h were not that significant, which was understandable given the short period after leonurine administration.

**FIGURE 1:**
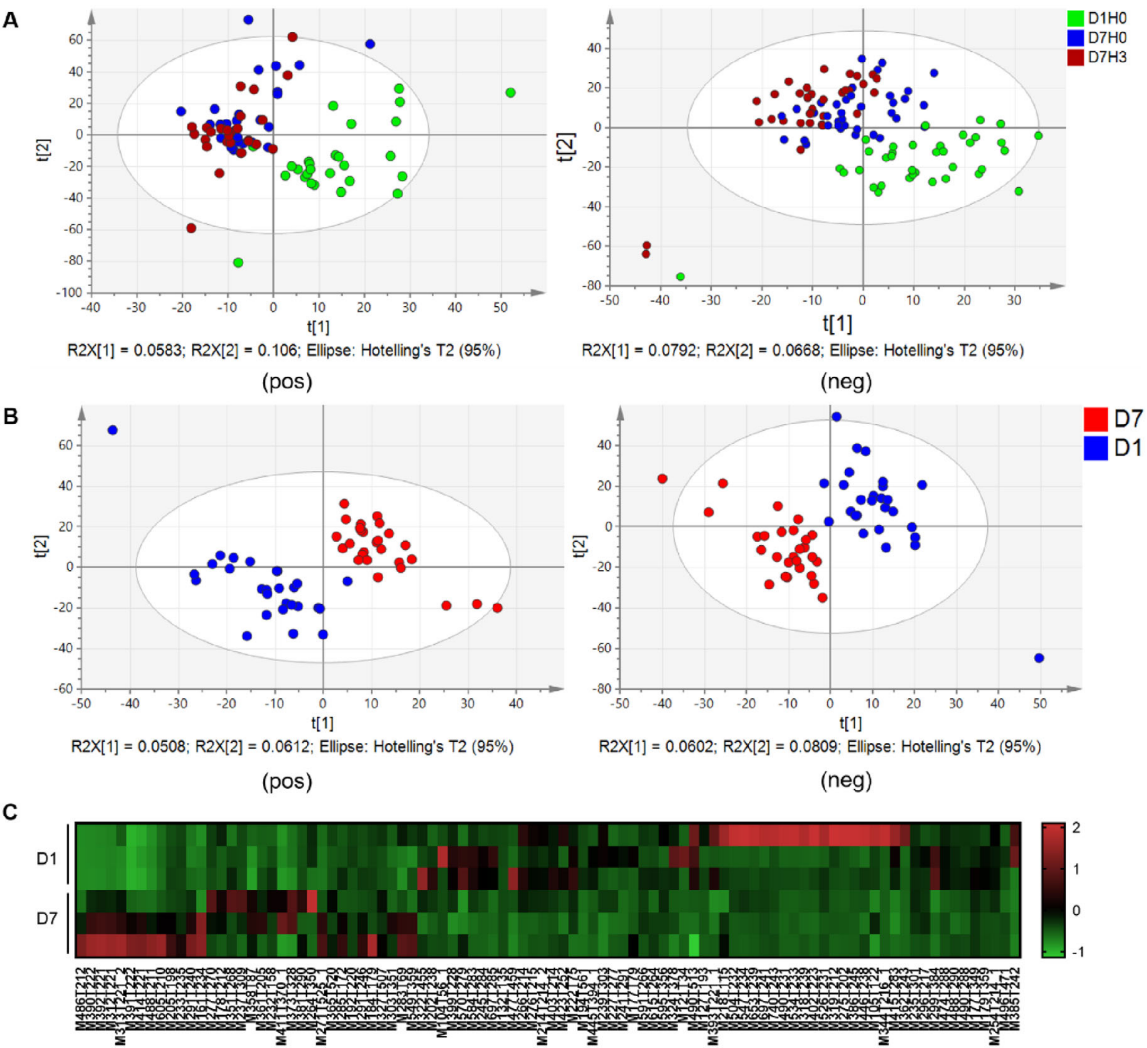
Multivariable analysis results among groups. (A) PLS‐DA score plots of detected metabolites in groups including D1‐0h, D7‐0h, and D7‐3h, in both positive and negative ion mode. (B) PLS‐DA score plots of detected metabolites in D1 and D7 (D7‐0h) in both positive and negative modes. (C) Heatmap of significant metabolites indicating differences between group D1 and D7

We then mainly focused on the group differences between D1 (D1‐0h) and D7 (D7‐0h). PLS‐DA was also applied to suggest the group differences (Figure 1B) in both positive (*R* ^2^ *_X_* = 0.328, *R* ^2^ *_Y_* = 0.994, *Q* ^2^ = 0.738) and negative ion mode (*R* ^2^ *_X_* = 0.232, *R* ^2^ *_Y_* = 0.990, *Q* ^2^ = 0.728). PLS‐DA also provided the parameter, variable importance in projection (VIP) to quantify the contribution of each metabolite to the group differences. Using fold change (D7/D1) higher than 2 or lower than 0.5, VIP > 1, and *P* < 0.05, we were able to locate the significant metabolites. In total, 94 significant metabolites were identified, with 48 in positive ion mode (15 increased and 33 decreased) and 46 in negative ion mode (19 increased and 27 decreased) as shown in the heatmap in Figure 1C. The details of these 94 metabolites can be seen in Supplement Tables.

Based on the significant metabolites, pathway analysis was performed by MetaboAnalyst.^10^ Pathway analysis suggested that cysteine and methionine metabolism could be affected by 7‐day leonurine treatment (Figure 2A). We then looked into the levels of metabolites involved in cysteine and methionine metabolism. The levels of methionine, one of the key metabolites, were slightly elevated by leonurine treatment (Figure 2B).

**FIGURE 2:**
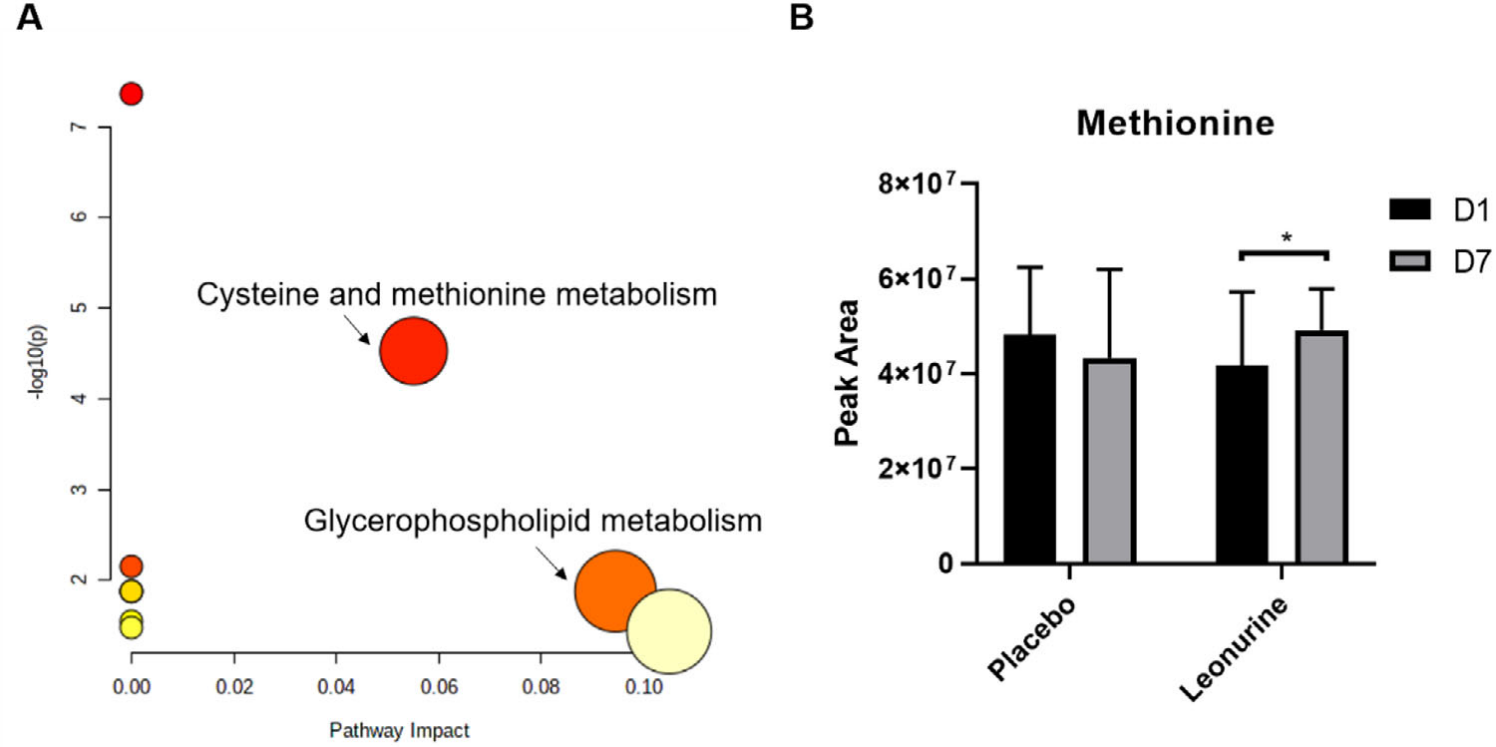
Pathway analysis based on significant metabolites. (A) Pathway analysis suggested that cysteine and methionine metabolism and glycerophospholipid metabolism could be affected by leonurine treatment. (B) Methionine levels were affected by leonurine treatment. Statistical analysis was performed by paired Student's *t*‐test, and * suggested that *P* value was lower than 0.05

16S ribosomal DNA (rDNA) sequencing was applied to study the gut microbiota changes after 7 days of leonurine treatment. Results suggested that the gut microbiota structure was altered by 7‐day leonurine treatment in both phylum (Figure 3A) and genus levels (Figure 3B). Linear discriminant analysis Effect Size (LEfSe) was then applied to identify the significantly altered bacteria before and after leonurine treatment (Figure 3C, D). At the genus level, the relative abundance of *Ruminococcus*, *Streptococcaceae*, and so on were elevated by leonurine treatment, while the relative abundance of *Myobacterium*, *Veillonella*, *Lachnospiraceae*, and *Weissella* were downregulated.

**FIGURE 3:**
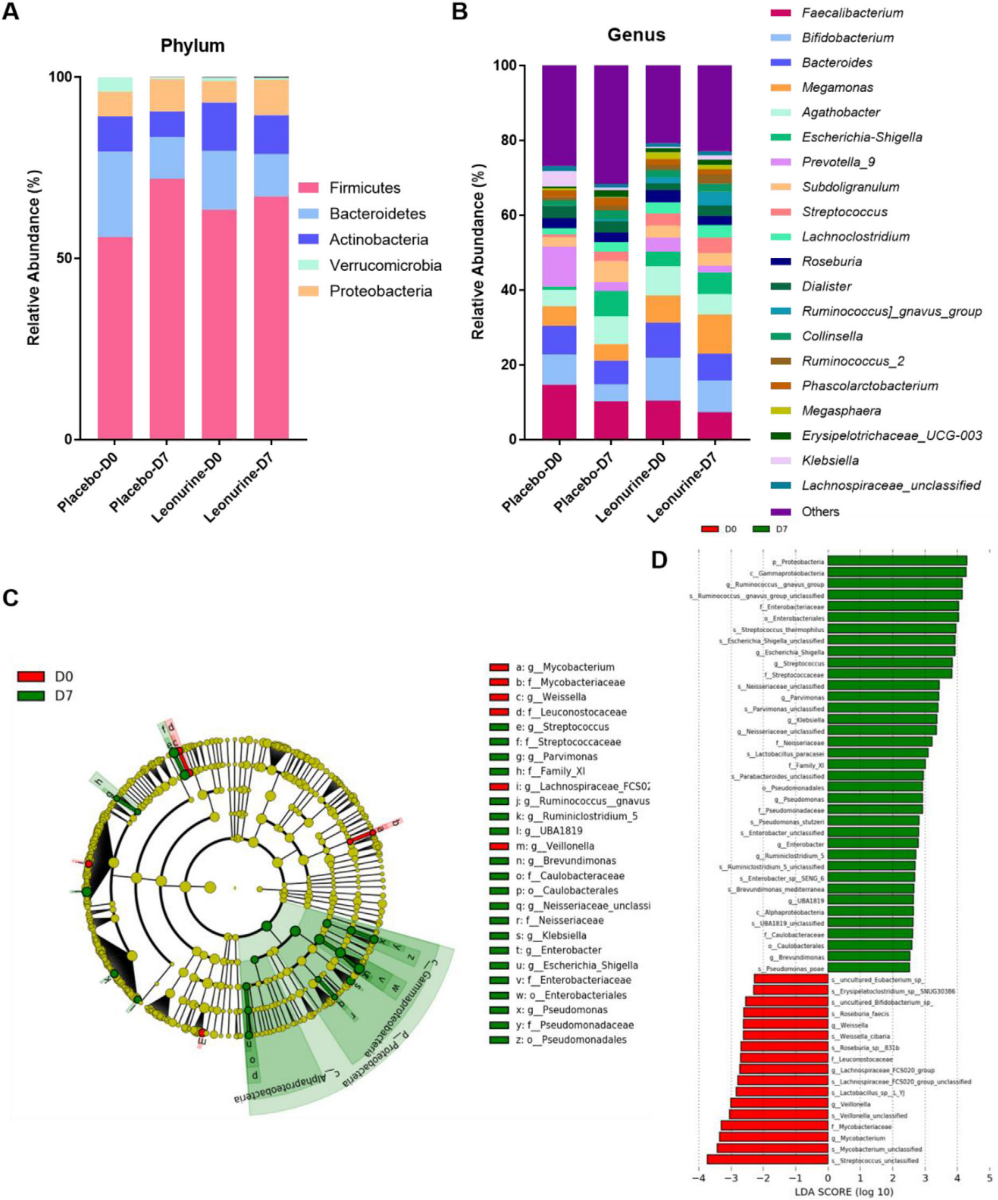
Gut microbiota structure before and after leonurine treatment. (A) The relative bacteria abundance in phylum level (5 highest phyla). (B) The relative bacteria abundance in genus level (20 highest genera). (C) LEfSe analysis and (D) LDA score suggested the significantly altered bacteria after leonurine treatment

Using the 16S rDNA sequencing results, we also did the functional prediction based on PICRUSt, connecting the altered gut microbiota structure with metabolism pathways (Figure 4). Most of the predicted metabolism pathways were upregulated. One of them, PWY‐5507, caught our attention. PWY‐5507 is also known as the adenosylcobalamin (AdoCbl) biosynthesis I (early cobalt insertion). AdoCbl is one of the active forms of the metabolite, cobalamin, which is an essential coenzyme for methionine synthase.

**FIGURE 4:**
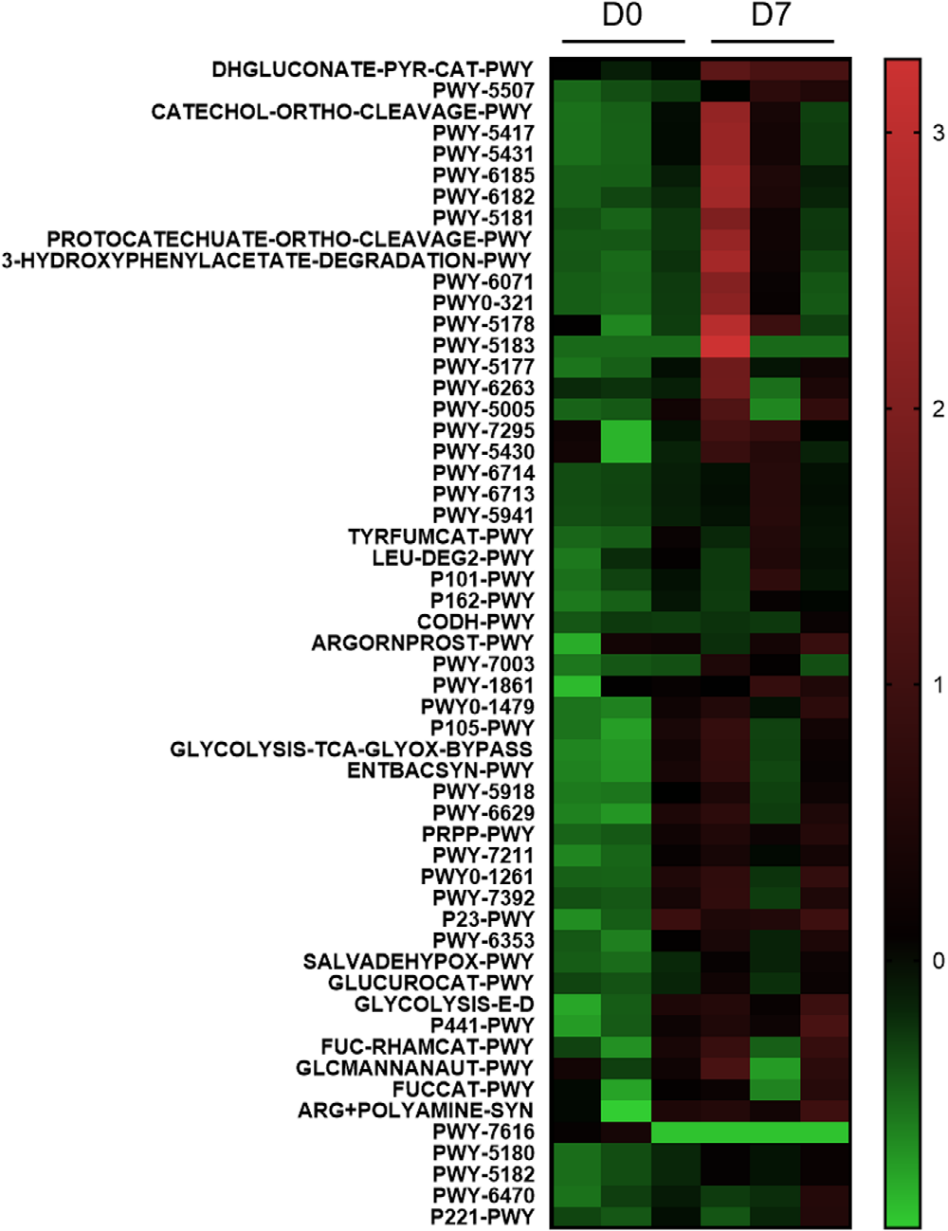
Functional prediction by PICRUST based on 16S rDNA sequencing results

In conclusion, combining the results from metabolomics and gut microbiota study, we proposed that leonurine could affect the gut microbiota structure, upregulating the biosynthesis of AdoCbl, and promoting the reaction converting homocysteine into methionine, which could be part of the mechanism explaining its cardioprotective effects. This study provided new insights into the mechanisms of leonurine and potential directions for future clinical trials.

## CONFLICT OF INTEREST

The authors claim that the researchers in this study have no conflict of interest.

## ETHICS APPROVAL AND CONSENT TO PARTICIPATE

The randomized double‐blinded leonurine phase I clinical trial was carried out in collaboration with Peking University First Hospital (Registration No. CTR20191574), with permission from Peking University First Hospital Drug Clinical Trial Ethics Committee. Participants admitted to the clinical trial were fully informed of the purpose and process of the clinical trial. And consent forms were signed.

## AUTHOR CONTRIBUTIONS

Yi Zhun Zhu and Yimin Cui conceived and designed the study and supervised the project. Junyi Liao, Rinkiko Suguro, and Xia Zhao collected and prepared the samples for analysis. Junyi Liao performed the analyses and interpreted the data. Junyi Liao and Yue Yu drafted the manuscript, and all of the authors revised the manuscript.

## Supporting information

Table S1

Table S2

## Data Availability

The data reported in this article will be made available for 5 years after publication with permission from the corresponding authors. Researcher will be required to provide a proposal and a signed data access agreement.

## References

[1] Ashfield‐Watt PA , Moat SJ , Doshi SN , McDowell IF . Folate, homocysteine, endothelial function and cardiovascular disease. What is the link? Biomed Pharmacother. 2001;55:425‐433. 11686575 10.1016/s0753-3322(01)00125-1

[2] Splaver A , Lamas GA , Hennekens CH . Homocysteine and cardiovascular disease: biological mechanisms, observational epidemiology, and the need for randomized trials. Am Heart J. 2004;148:34‐40. 15215789 10.1016/j.ahj.2004.02.004

[3] Jakubowski H . Homocysteine modification in protein structure/function and human disease. Physiol Rev. 2019;99:555‐604. 30427275 10.1152/physrev.00003.2018

[4] Jang C , Chen L , Rabinowitz JD . Metabolomics and isotope tracing. Cell. 2018;173:822‐837. 29727671 10.1016/j.cell.2018.03.055 PMC6034115

[5] McGarrah RW , Crown SB , Zhang GF , Shah SH , Newgard CB . Cardiovascular metabolomics. Circ Res. 2018;122:1238‐1258. 29700070 10.1161/CIRCRESAHA.117.311002 PMC6029726

[6] Dabke K , Hendrick G , Devkota S . The gut microbiome and metabolic syndrome. J Clin Invest. 2019;129:4050‐4057. 31573550 10.1172/JCI129194 PMC6763239

[7] Thursby E Juge N . Introduction to the human gut microbiota. Biochem J. 2017;474:1823‐1836. 28512250 10.1042/BCJ20160510 PMC5433529

[8] Zhu YZ , Wu W , Zhu Q , Liu X . Discovery of Leonuri and therapeutical applications: from bench to bedside. Pharmacol Ther. 2018;188:26‐35. 29360539 10.1016/j.pharmthera.2018.01.006

[9] Suguro R , Chen S , Yang D , et al. Anti‐hypercholesterolemic effects and a good safety profile of SCM‐198 in animals: from ApoE knockout mice to rhesus monkeys. Front Pharmacol. 2018;9:1468. 30618759 10.3389/fphar.2018.01468 PMC6300478

[10] Chong J , Wishart DS , Xia J . Using MetaboAnalyst 4.0 for comprehensive and integrative metabolomics data analysis. Curr Protoc Bioinformatics. 2019;68:e86. 31756036 10.1002/cpbi.86

